# Supplementary material for: Myonuclear alterations associated with exercise are independent of age in humans
Source: J Physiol. 2023 Jan 30;603(13):3755–75. doi: 10.1113/JP284128 (PMC12306410; doi:10.1113/JP284128)
Supplement: Supplementary file 1 — Statistical Summary Document [file TJP-603-3755-s001.pdf]

The Journal of Physiology

Statistical Summary Document

Manuscript Title: Myonuclear alterations associated with exercise are independent of age in humans

Authors: Edmund Battey, Jacob A Ross, Anthony Hoang, Darren G S Wilson, Yu Han, Yotam Levy, Ross D Pollock, Michaeljohn Kalakoutis, Jamie Pugh, Graeme L Close, Georgina M Ellison, Norman R Lazarus, Thomas Iskratsch, Stephen D. H. Harridge, Julien Ochala, and Matthew J Stroud

Animal model used, if applicable: Human – volunteer participants. Mouse – C57BL/6 wild-type.

Underlying hypothesis: This investigation tests the hypothesis that exercise affects the shape and mechanical properties of skeletal muscle nuclei.

Definitions of ‘n’:

Question 1: n = number of human volunteers per group
Question 2: n = number of animals of the specified conditions
Question 3: number of animals of the specified conditions

The Journal of Physiology Statistical Summary Document

Statistical summary table:

Experimental question number*	Finding/ conclusion	Experimental location/ variable e.g. muscle, neocortex or genotype	Mean values (or other summary statistic)	Standard deviation	n val.	P**	Units	Data comparisons e.g. WT vs KO	Statistical test	Any other variable e.g. subjects' age or sex	Figure/ table in which data are presented	Comments e.g. observation
1. Nuclear shape changes?	Reduced aspect ratio in YT and OT compared to YU and OU	Skeletal muscle	27-29% reduction in aspect ratio in trained groups YU, 2.4; OU, 2.3; YT, 1.7; OT, 1.6	YU: 0.3 OU: 0.3 YT: 0.1 OT: 0.2	6 per group	<0.0001	N/A (aspect ratio is length/width)	YU vs YT vs OU vs OT	Two-way ANOVA Tukey's Multi. comp.	Mixed sex groups	1	
2. Differences in the expression of nuclear envelope/ nuclear lamina proteins?	Greater lamina deposition in OT compared to OU	Skeletal muscle	OU: 0.25 OT: 0.30	OU: 0.015 OT: 0.021	OU: 5 OT: 3	0.0168	AU	OU vs OT	Unpaired-T-test	Mixed sex groups	3	

	Higher Lamin A levels in exercise-trained compared mice to untrained mice	Skeletal muscle	Trained: 442 Untrained: 384	Trained: 53 Untrained: 33	7	-	AU	Trained vs untrained	Unpaired-T-test	-	4	
3 Altered nuclear mechanics?	More deformable nuclei in OU compared to OT	Skeletal muscle	R values for correlation between aspect ratio and sarcomere length were 0.36 and 0.45 for OT and OU, respectively Slopes of correlation between sarcomere length and aspect ratio were 0.79 and 0.29 in OU and OT, respectively	-	3	Correlation <0.0001 Difference between slopes <0.0001	N/A	OU vs OT	Simple linear regression was performed to test if slopes were significantly non-zero and nonlinear straight-line regression analyses were performed to compare the slopes	Mixed sex groups	5	

									of different conditions.			
	Stiffer nuclei in trained compared to untrained mice	Skeletal muscle	Trained: 1.7<br>Untrained: 3.2	Trained: 0.9 Untrained: 1.3	3	0.0393	kPa	Trained vs untrained	Paired t-test	-	5	
	Altered viscoelasticity of nuclei in trained mice		Tan δ was on average ~20% lower in trained mice compared to untrained mice at 1, 2 and 4 Hz DMA.<br>Overall means, Trained: 0.18; Untrained 0.22<br>Means at 1 Hz, Trained: 0.154; Untrained: 0.1922<br>Means at 2 Hz, Trained:	Trained: 0.03; Untrained: 0.03	3	0.0193 (1 Hz)<br>0.0381 (2 Hz)<br>0.0262 (4 Hz)<br>0.0989<br>(10 Hz)	N/A (Tan δ is E’/E’’)	Trained vs untrained	Mixed effects analysis (Sidak’s multiple comparison test)	N/A	5	

			0.163; Untrained: 0.2035<br><br>Means at 4 Hz, Trained: 0.178; Untrained: 0.225<br><br>Means at 10 Hz, Trained: 0.255; Untrained: 0.261									

*You may use multiple lines for the same question to indicate multiple comparisons

** Authors may wish to make the text bold where p is considered significant against a stated confidence limit.
